# Post-Transcriptional Regulation of *Gnrhr*: A Checkpoint for Metabolic Control of Female Reproduction

**DOI:** 10.3390/ijms22073312

**Published:** 2021-03-24

**Authors:** Angela K. Odle, Melanie C. MacNicol, Gwen V. Childs, Angus M. MacNicol

**Affiliations:** Department of Neurobiology and Developmental Sciences, University of Arkansas for Medical Sciences, Little Rock, AR 72205, USA; MacnicolMelanie@uams.edu (M.C.M.); ChildsGwenV@uams.edu (G.V.C.); Angus@uams.edu (A.M.M.)

**Keywords:** gonadotropin-releasing hormone receptor, post-transcriptional control, leptin

## Abstract

The proper expression of gonadotropin-releasing hormone receptors (GnRHRs) by pituitary gonadotropes is critical for maintaining maximum reproductive capacity. GnRH receptor expression must be tightly regulated in order to maintain the normal pattern of expression through the estrous cycle in rodents, which is believed to be important for interpreting the finely tuned pulses of GnRH from the hypothalamus. Much work has shown that *Gnrhr* expression is heavily regulated at the level of transcription. However, researchers have also discovered that *Gnrhr* is regulated post-transcriptionally. This review will discuss how RNA-binding proteins and microRNAs may play critical roles in the regulation of GnRHR expression. We will also discuss how these post-transcriptional regulators may themselves be affected by metabolic cues, specifically with regards to the adipokine leptin. All together, we present evidence that *Gnrhr* is regulated post-transcriptionally, and that this concept must be further explored in order to fully understand the complex nature of this receptor.

## 1. Introduction

The hypothalamic–pituitary–gonadal (HPG) axis is a finely tuned, dynamic system that is heavily influenced by environmental factors. The female HPG axis is especially remarkable in that it is programmed to cycle every 30 days in humans [1] and every 4–6 days in mice [2] and rats [3] in order to maintain follicular development and maturation in preparation for conception. Indeed, researchers have found that many factors in the HPG axis “cycle” in the female, involving layer after layer of sophisticated regulation [4]. The cells within the pituitary that respond to hypothalamic and gonadal factors to maintain these reproductive cycles are the gonadotropes. Although they make up a relatively small percentage of the anterior pituitary, the gonadotropes are a powerful population of cells that must function properly in order for reproductive capacity to be maintained [5]. Gonadotropes are an extremely dynamic population of cells that are poised to discern discreet patterns of gonadotropin-releasing hormone (GnRH) secretion from the hypothalamus [6,7,8,9,10,11,12]. These GnRH pulses are critical for directing the production and/or secretion of basal and stimulated levels of gonadotropins that subsequently control reproductive processes. Arguably, one of the most important products of the gonadotrope is the gonadotropin-releasing hormone receptor (GnRHR), the expression of which is necessary for the receipt and interpretation of GnRH pulses [6]. 

The GnRHR is a member of the G protein-coupled receptors and is one of the smallest receptors of this class in mammals [13]. GnRHR levels are considered to be the rate-limiting component of gonadotrope functions. Indeed, proper levels of the receptor are necessary for maintaining the production of the major gonadotrope products follicle-stimulating hormone (FSH) and luteinizing hormone (LH), and the concentration of GnRH receptors on the gonadotrope surface may actually dictate the preferential stimulation of gonadotropin transcription [6]. A seminal study by Savoy-Moore et al. detailed the pattern of GnRHR expression in the female rat pituitary through the estrous cycle using radiolabeled GnRH agonists [14]. The rat pituitary displays a significant increase in GnRH receptors from metestrus to diestrus, maintaining this high expression into the morning of proestrus, after which receptor expression rapidly declines. This study and others revealed that, in addition to changes occurring in GnRH pulses coming from the hypothalamus, GnRH receptor expression itself changes during the estrous cycle to prepare for the proestrous luteinizing hormone surge [15,16]. Thus began a decades-long investigation into the mechanisms behind regulation of GnRHR levels. 

Although many of the studies investigating the regulation of GnRH receptor expression have focused on the gonadotrope, GnRH receptors are also found in the gonads [17,18,19], placenta [20], spinal cord [21], and brain (hippocampus, amygdala, septum, hypothalamus, and cortex) [22,23,24,25,26], as well as in other tissues in various species. For this review, we will focus on the regulation of GnRH receptor expression in the pituitary. Most of the research to date has focused on the transcriptional regulation of the *Gnrhr* gene. However, in this review we will discuss emerging evidence that GnRHR levels are regulated post-transcriptionally, and that organismal metabolic status may heavily influence this regulation. Most of this evidence comes from studies of gonadotrope cell lines and/or female mice, so this review will focus largely on female reproduction. 

## 2. Transcriptional Regulation of *Gnrhr*

**Figure 1:**
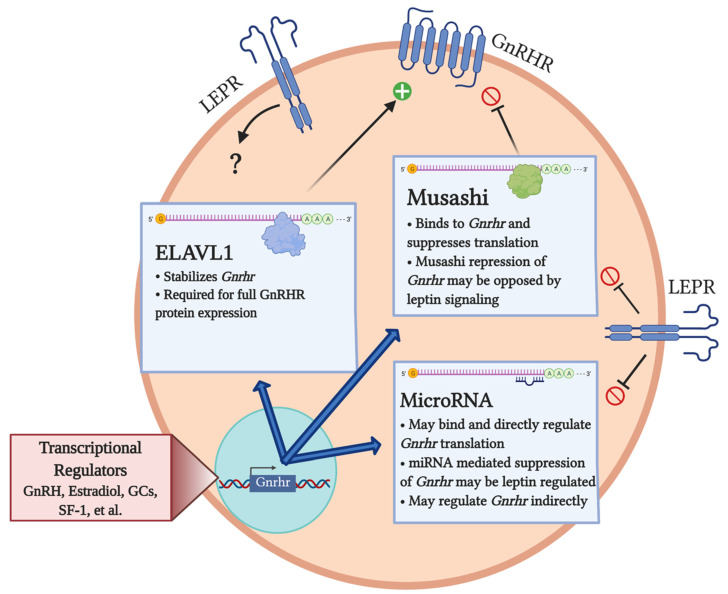
**Post-transcriptional control of *Gnrhr* and the role of leptin.** The proposed mechanisms for post-transcriptional control of *Gnrhr* are depicted, with leptin negatively regulating Musashi and microRNAs. The role of leptin in the regulation of ELAVL1 (if any) is unknown. Abbreviations: embryonic lethal abnormal vision like 1 (ELAVL1), leptin receptor (LEPR), glucocorticoids (GCs), steroidogenic factor-1 (SF-1).

The transcriptional regulation of the *Gnrhr* gene is undoubtedly critical for the fully functional gonadotrope, and the nature of this regulation has been reviewed extensively [12,27,28,29]. In brief, it is well established that transcription of *Gnrhr* is stimulated by GnRH, and that regulatory elements for GnRH-mediated stimulation are contained within the 5′ region of the murine *Gnrhr* [12,30,31,32]. Activin, a product of the ovaries and of the pituitary itself that is known to potently stimulate *Fshb* gene transcription, was once believed to amplify GnRH-stimulated *Gnrhr* transcription [33,34]. However, recent evidence has called this into question [35,36]. Other studies have shown that estradiol [30,37] and glucocorticoids [38] can stimulate *Gnrhr* transcription. Pituitary adenylate cyclase-activating polypeptide (PACAP) and steroidogenic factor-1 (SF-1) have been identified as important mediators of *Gnrhr* transcription [39,40,41], as has liver receptor homolog-1 (LRH-1) [42]. This list is not exhaustive, and *Gnrhr* is clearly highly regulated at the level of transcription, perhaps most significantly by GnRH itself. Newer evidence, however, has emerged indicating that regulation of this critical receptor extends well beyond transcription (Figure 1). 

## 3. Post-Transcriptional Regulation of *Gnrhr*

Discreet GnRH pulses increase the numbers of GnRH receptors in gonadotropes [43,44]. Although a great deal of GnRH-mediated stimulation of GnRHR exists at the level of transcription, GnRHR levels are also translationally regulated by GnRH. Studies from the mid-1990s showed that GnRH stimulates GnRHR protein levels without altering *Gnrhr* mRNA levels in the alpha T-3 cell line and in *Xenopus* oocytes [45]. The authors of this study suggested that the synthesis of GnRHR can be regulated at both transcriptional and post-transcriptional levels, depending on variables such as time and species. A second study by the same group demonstrated that the decrease in GnRHR following chronic stimulation with GnRH in alpha T3 cells is due to a decrease in *Gnrhr* mRNA translation [46]. Researchers therefore began exploring a new level of *Gnrhr* regulation, focusing on post-transcriptional mechanisms. We review here three mechanisms by which *Gnrhr* is regulated post-transcriptionally (Figure 1).

### 3.1. miRNA Regulation of Gnrhr

MicroRNAs (miRNA) are single-stranded RNAs (~22 nucleotides) that bind and regulate target RNAs, leading to changes in translation or stability. The expression and function of gonadotrope miRNAs has been studied in LßT2 cells [47,48,49,50], rats [51,52], pigs [53,54], and mice [55,56,57]. Dicer is an endoribonuclease critical for the production of miRNAs from double-stranded mRNAs [58]. A gonadotrope-specific deletion of Dicer in mice has significant negative reproductive consequences [57]. Pituitaries from male gonadotrope-*Dicer*-null mice express much lower levels of the gonadotropin subunit mRNAs *Fshb* and *Lhb*, as well as the shared alpha (*Cga*) subunit mRNA compared to controls, and females also show a significant decrease in *Lhb* and *Cga* (but not *Fshb*). Importantly, both males and females from this gonadotrope Dicer-null line show significant decreases in pituitary *Gnrhr* mRNA. Thus, the authors proposed that this decrease in *Gnrhr* mRNA is the result of imbalances in gonadotrope transcriptional suppressors and activators following the loss of DICER-dependent miRNAs (see Figure 1 in [57]). Based on in silico predictions, *Gnrhr* mRNA may also be targeted by a number of miRNAs (Table 1, [59]). Potential regulatory miRNAs include miR-669d-5p and miR-3061-3p, which are predicted to target both human and mouse *Gnrhr* 3′ untranslated regions (UTRs) [4].

Table 1: Targetscan 7.2-predicted miRNA target sites within the 190 nucleotide mouse *Gnrhr* 3′ UTR (ENST00000420975 (nucleotides 110–299)) [59,60]. The position of each miRNA target site is expressed as nucleotides from the 5′ end of the *mGnrhr* 3′ UTR. Target site conservation within either the human or rat *Gnrhr* 3′ UTR is indicated. For reference, our cloned 190 nucleotide mouse *Gnrhr* 3′ UTR contains three consensus Musashi binding elements (MBEs) (at positions 25–29, 106–111 and 143–148) as well as two atypical polyadenylation hexanucleotide sequences (AACAAA) at 174–179 and 181–186 [60].

While researchers are still determining which microRNAs are expressed in gonadotropes and how miRs may regulate gonadotrope functions, there have been several studies exploring the responses of gonadotrope miRNA to GnRH. An initial survey of miRNAs in LßT2 cells using multiple detection methods revealed a large number of miRNAs expressed in this gonadotrope cell line, with the exact number depending on the sensitivity of the method [47]. A test of GnRH responsiveness revealed selective upregulation of miR-132/miR-212 (products of the same gene). A second study of miRNAs in the LßT2 cell line demonstrated that GnRH stimulates both an up- and down-regulation of gonadotrope miRNAs, confirming a strong upregulation of miR-132/miR-212 [48]. A focused investigation of miR-132 and miR-125b revealed a regulatory loop important for the desensitization of the gonadotrope to the GnRH signal, though no direct interaction with the *Gnrhr* transcript was discussed [50]. Rather, the authors found several downstream targets for both miRNAs that appear to be critical for proper gonadotrope responses to GnRH. The same group has also identified a role for miR-132/212 in GnRH-mediated stimulation of *Fshb* expression [49]. GnRH stimulation of primary porcine pituitary cell cultures also revealed both up- and down-regulated miRNAs, including miR-361-3p (downregulated) which the authors state is predicted to bind *Gnrhr* 3′ UTR [53]. We note that Targetscan did not predict binding of miR-361-3p to the murine *Gnrhr* 3′ UTR (Table 1). While the role and species-specific identity of miRNAs that may directly regulate *Gnrhr* mRNA translation remain to be fully determined, it is clear that miRNAs make a significant contribution to the transcriptional and/or post-transcriptional homeostasis of the gonadotrope in vivo [57]. Future studies of gonadotrope-specific miRNA modulations following physiological stimulations and targeted analyses of their ability to modulate *Gnrhr* mRNA translation and stability will be critical to clarify their regulatory impact.

### 3.2. Post-Transcriptional Regulation of Gnrhr mRNA by Musashi

An *in silico* analysis of the *Gnrhr* mRNA regulatory 3′ untranslated region (3′UTR) by our laboratory revealed three consensus binding sites for the RNA-binding protein Musashi [60]. The Musashi gene family (*Msi1* and *Msi2*) encodes sequence-specific regulators of target mRNA translation that have been implicated as critical determinants of stem cell fate [61]. In addition to pituitary stem cell expression, our recent work has found expression of both *Msi1* and *Msi2* throughout all hormone-producing cell lineages of the anterior pituitary [62] where they may function to modulate hormone secretion in response to organismal demands. 

Using an electrophoretic mobility shift assay (EMSA), we showed that the Musashi1 protein (MSI1) interacts specifically with the murine *Gnrhr* 3′UTR [60]. To test if MSI1 association enforced translational regulation through the *Gnrhr* 3′ UTR, we performed a series of dual luciferase mRNA reporter assays. We reported that MSI1 significantly repressed the translation of the *Gnrhr* 3′ UTR reporter mRNA [60]. Importantly, immunoprecipitation of MSI1 from female mouse pituitaries significantly enriched for the endogenous *Gnrhr* mRNA, confirming this relationship in the mouse pituitary. Not only did these studies identify a new mechanism for the regulation of GnRHR levels in the mouse, they also proposed a novel role for MSI in the regulation of mature, hormone-secreting pituitary cells. It will be important to define other MSI targets in these cells. As the levels of GnRHR change throughout the estrous cycle [14], it will also be important to determine if MSI plays a role in the stage-dependent expression of these receptors. 

### 3.3. Post-Transcriptional Regulation of Gnrhr by ELAVL1

A second RNA-binding protein, embryonic lethal abnormal vision like 1 (ELAVL1/HuR), has been implicated as a post-transcriptional regulator of *Gnrhr* mRNA [63]. ELAVL1 is a member of the ELAV/Hu family of RNA-binding proteins that are known for their ability to bind AU-rich elements (AREs) to stabilize mRNA transcripts and thus promote translation [64]. In LßT2 cells, a gonadotrope model cell line, immunoprecipitation of ELAVL1 revealed a number of direct mRNA targets that are critical for gonadotropin synthesis and release, including the *Gnrhr* mRNA. Knockdown of the *Elavl1* gene in these cells decreases *Gnrhr* mRNA significantly. Importantly, ELAVL1 binding was not altered by GnRH stimulation, although ELAVL1 interaction with certain other mRNAs was altered. The authors showed that ELAVL1 functions to stabilize *Gnrhr* mRNA, and that this stabilization is critical for full GnRHR protein expression. Since several other important gonadotrope genes were also associated with ELAVL1, this study suggests a broader role for ELAVL1 in regulating gonadotrope mRNA stability and translation.

## 4. Metabolic Influences on the Post-Transcriptional Regulation of GnRHR Levels: A Role for Leptin

The negative repercussions of nutritional unbalance, including obesity, high-fat diet, and caloric insufficiency upon reproduction are numerous. At the level of the pituitary, we know that diet-induced obesity (DIO) in mice can cause increased GnRH receptor levels and inappropriately high GnRH-stimulated LH secretion, similar to what is seen in polycystic ovarian syndrome (PCOS) [65,66]. While it is possible that several circulating metabolic factors are involved, most of what we currently know about the effects of metabolism on the post-transcriptional regulation of *Gnrhr* comes from studies of leptin. Leptin is an adipokine produced by white fat cells that signals the body’s nutritional status to nearly every tissue in the body, including each tier of the HPG axis [67]. Leptin circulates in proportion to fat, such that lean individuals have normal or low-circulating leptin, whereas obese individuals have high-circulating leptin [68,69]. Maintaining levels of circulating leptin within a narrow optimal range is critical, as having low- or high-circulating leptin can lead to reproductive disorders [70,71,72]. For example, women with PCOS have been shown to have decreased leptin-LH pulse synchronization compared to control subjects [73]. Female (and most male) mice lacking the leptin gene (*Lep/Lep*) or leptin receptors (*Lepr/Lepr*) are infertile [74,75,76], and administration of leptin to *Lep/Lep* mice can restore fertility without a significant decrease in weight [77,78]. Importantly, all three tiers of the hypothalamic–pituitary–gonadal (HPG) axis express leptin receptors (LEPR) [67,79,80,81]. While much attention has been given to the important role leptin plays in the brain, work from our lab has revealed novel mechanisms by which leptin regulates reproduction at the level of the pituitary.

As mentioned above, gonadotropes must produce sufficient and appropriate amounts of GnRHR to maintain reproductive functions. Just as GnRH receptor levels change with the estrous cycle in rodents, so do the levels of gonadotrope LEPR. Our lab showed that, in both rats and mice, the overall percentage of pituitary cells expressing leptin receptors is significantly higher in proestrus and estrus compared to metestrus and diestrus [82]. When focusing on the gonadotropes, cells co-expressing LH and LEPR or FSH and LEPR increase from metestrus to diestrus, remaining high in proestrus decreasing by estrus. Also increasing from metestrus to diestrus are GnRHR proteins, suggesting that the increase in gonadotrope leptin sensitivity may indeed be needed to stimulate the cyclic rise in GnRHR. 

In order to study the role of leptin in the regulation of GnRHR, we produced a gonadotrope-specific *Lepr* knockout line [82]. We found that leptin receptors on gonadotropes are important for maximal reproductive competence, perhaps due to the reduced gonadotropin secretion seen in these female mice. A study of the pituitaries from these sub-fertile female gonadotrope *Lepr*-null mice revealed decreased GnRH receptor proteins with no change in *Gnrhr* mRNA [82]. We therefore hypothesized that leptin regulates gonadotrope functions, at least in part, by regulating *Gnrhr* at the post-transcriptional level.

We have demonstrated that MSI1 binds to *Gnrhr* in the mouse pituitary [60]. Leptin appears to exert a regulatory influence on this relationship as leptin stimulation of normal pituitary cells decreases *Msi1* expression in vitro. In separate studies, we also showed that leptin can oppose MSI-mediated mRNA translational repression of the *Pou1f1* (pituitary-specific positive transcription factor 1) mRNA 3′ UTR in reporter assays [62]. Stimulation of primary pituitary cultures with recombinant mouse leptin resulted in an increase in GnRH binding sites and a decrease in MSI1 [60], supporting the hypothesis that leptin acts to oppose Musashi-directed repression of the *Gnrhr* mRNA in gonadotropes. Our ongoing studies will determine if, like *Pou1f1* mRNA translation [62], leptin acts to oppose MSI-mediated repression to promote *Gnrhr* mRNA translation in gonadotropes. 

Metabolic disorders are associated with disordered levels of miRNA production and secretion (reviewed in [83]). Specifically, leptin has been shown to modulate miRNA levels (see [84,85,86,87] for a few recent examples), and we have demonstrated this in somatotropes [88]. Since our in silico analyses predicted binding sites for miRNAs within the 3′UTR of *Gnrhr* (Table 1), we wished to determine whether or not leptin may regulate *Gnrhr* post-transcriptionally via regulation of gonadotrope miRNA levels. To this end, we selected miRNAs from our in silico analysis and measured them in our control and gonadotrope-leptin receptor-null female mouse pituitaries. One of these miRNAs, miR-669d, was found to be significantly increased in our gonadotrope-*Lepr*-null female pituitaries [4], consistent with the hypothesis that leptin downregulates gonadotrope miRNAs that can target the *Gnrhr* mRNA for repression. Future studies characterizing the gonadotrope microtranscriptome before and after leptin stimulation will determine if any of the miRNAs predicted to bind *Gnrhr* are metabolically sensitive. We also recognize that circulating miRNAs, which are altered with metabolic status, may play a role in *Gnrhr* regulation. Indeed, researchers have previously identified two miRNAs critical for LH secretion [89] that are dysregulated in the serum of anovulatory women [90]. 

## 5. Future Directions: Developmental Regulation of GnRHR

The *Gnrhr* transcript is one of the earliest detectable gonadotrope markers, first appearing around embryonic day 12.75–13.5 in mice [91,92]. The embryonic mouse gonadotropes have been shown to respond to GnRH by increasing secretion of LH on embryonic day 16 [92,93]. A study of male mice in which yellow fluorescent protein (YFP) is expressed in *Gnrhr*-expressing cells demonstrated that all LHß+ cells express GnRHR by embryonic day 16.5, whereas FSHß+ cells begin to express GnRHR around embryonic day 18.5, with full expression (all FSHß+ cells expressing GnRHR) not seen until postnatal day 7 (44). GnRH signals from the hypothalamus continue to activate gonadotropes into the postnatal days (rodents) and months (humans) (reviewed in [94]). Finally, at the initiation of puberty, GnRH pulse amplitude and frequency begin increasing, and male and female patterns of GnRH secretion are ultimately established [94]. Therefore, the proper expression of GnRH receptors during the embryonic and postnatal periods is critical for the development and refinement of the HPG axis. GnRH receptor transcript levels rise sharply in the postnatal weeks in rats, peaking before (male) or right around (female) the onset of puberty and declining into adulthood [95].

Also occurring during this postnatal period in rodents (often considered equivalent to the third-trimester in humans) is the remarkable postnatal leptin surge [96,97]. In rodents, this surge in leptin secretion occurs between PND 7 and 10, returns to adult levels by weaning, and is not associated with increased food intake or fat accumulation. Full and properly timed execution of the postnatal leptin surge has been found to be critical for the normal development of the pancreas, the kidney, the thymus, the ovaries, and hypothalamic neuronal and glial populations involved in metabolism, in addition to other areas in the brain [98,99,100,101,102]. One study in male and female rats used a leptin antagonist to block the surge and looked at changes in puberty markers [103]. The investigators found increased estradiol and *Fshb* production, along with increased kisspeptin receptor in the treated prepubertal females, indicating that the onset of puberty is perhaps advanced in this group. These findings demonstrate the sensitivity of the reproductive axis to the postnatal leptin surge. Investigators must work to determine how the regulation of GnRH receptor expression is influenced by leptin during this critical postnatal period.

Regulation of GnRH receptor expression is multifaceted, involving both transcriptional and post-transcriptional mechanisms, and clearly more research is required to elucidate the molecular pathways influencing gonadotropin release and their interactions with the metabolic axis, not just in the adult, but also during embryonic development. Indeed, leptin is permissive for puberty, and exogenous leptin can advance the onset of puberty in normal female mice [104,105,106,107]. To what extent do embryonic post-transcriptional pathways establish reproductive competence for the adult? In the adult, future studies will be necessary to assess the ability of gonadotrope miRNAs to directly modulate *Gnrhr* mRNA translation and assess their regulation by leptin-dependent signaling. It will prove illuminating to determine if MSI and ELAVL1 share mRNA targets within the gonadotrope population and, if so, whether they act antagonistically, cooperatively or independently to govern gonadotropin release. It will also prove helpful to know if MSI is regulated by GnRH and, conversely, if ELAVL1 is a target of leptin signaling. In addition to the *Gnrhr* mRNA, it is important to determine if there are other gonadotrope mRNAs subject to post-transcriptional regulation if these modulate estrus cycle function and/or reproductive competence.

Previous studies have reported that in addition to ELAVL1/HuR, other AU-rich binding proteins (e.g., tristetrapolin (TTP)/zinc finger protein 36 (ZFP36)) can sometimes collaboratively or antagonistically bind to the same target mRNA to enhance or attenuate translational regulation [108]. It remains to be determined if TTP or other AU-rich binding proteins contribute to *Gnrhr* mRNA translational regulation. An additional complexity that will need to be considered is the possibility of collaborative or antagonistic RNA-binding protein/miRNA competition for overlapping 3′ UTR target sites, particularly with relation to ELAVL1/HuR [109]. Of note, several putative miRNAs that target the *Gnrhr* mRNA translation overlap the MBEs (Table 1), and so it may prove informative to determine if miRNAs influence Musashi-dependent regulation. The competition between RNA-binding proteins and/or RNA-binding proteins and miRNA for a common binding site represents an interesting future area of study. Given the interest in HuR inhibitors as cancer therapeutics [110,111,112], researchers should determine whether or not these drugs have negative effects on the HPG axis (such as the destabilization of *Gnrhr*) and if these effects are long-lasting. Lastly, much recent interest has been focused on the role of long noncoding RNAs (lncRNAs) in modulating mRNA translation and miRNA-dependent regulation [113]. While the repertoire of gonadotrope-expressed lncRNAs and their responses to leptin and/or GnRH signaling remain to be determined, lncRNAs may nonetheless be an additional level of direct or indirect control of GnRHR levels in the gonadotrope. Each of these potential regulators may translate into therapeutic targets for treating sub-optimal fertility. These potential RNA-binding protein, miRNA, and lncRNA regulatory pathways may ultimately be leveraged as novel therapeutic targets for treating sub-optimal fertility. Such strategies may, for example, be designed to suppress specific miRNAs [114] or promote ELAVL1/HuR stabilization of *Gnrhr*. 

## 6. Conclusions

For decades, researchers have worked to determine the pathways ultimately leading to the secretion of LH and FSH, critical mediators of reproduction. One of the most important stimulators of the gonadotropins is GnRH from the hypothalamus. Therefore, the regulation of the GnRH receptor is critical for reproduction. Years of elegant studies have shown that *Gnrhr* is transcriptionally regulated. However, less is known about post-transcriptional control. Older and more recent studies by our lab and others have uncovered new layers of regulation of GnRHR expression (Figure 1). Given the evidence that leptin, a critical metabolic signal, may regulate miRNAs and RNA-binding proteins targeting *Gnrhr* mRNA, the study of post-transcriptional regulation of *Gnrhr* may inform future therapies targeted at optimizing reproductive capacity in women with metabolic challenges. 

## Figures and Tables

**Table 1:** miRNAs predicted to bind the murine *Gnrhr* 3′ UTR.

Predicted 3′ UTR Target Site	Position in Mouse *Gnrhr* 3′ UTR (ntds)	Conserved in Human *Gnrhr* 3′ UTR?	Conserved in Rat *Gnrhr* 3′ UTR?	miRNA Target Site Overlap with MBE?
miR-150-5p	1–8			
miR-532-3p	2–8		Yes	
miR-669d-5p	11–18	Yes		
miR-3089-5p	17–23			
miR-1199-5p	19–25			Yes
miR-3061-3p	33–39	Yes		
miR-7223-5p	66–72			
miR-599	75–81			
miR-467eh-5p/miR-668-5p	79–85			
miR-344-3p/miR-410-3p	83–89			
miR-1981-3p	106–112			Yes
miR-495-3p	136–142		Yes	
miR-3065-5p	137–143		Yes	Yes
miR-493-3p	158–165		Yes	
miR-129-5p	176–182			
miR-129-5p	183–189			

## Data Availability

None.
